# Case Report: The “delta effect”: when endocrine pathology hides in plain sight

**DOI:** 10.3389/fendo.2026.1756544

**Published:** 2026-04-28

**Authors:** Philip J. G. M. Voets

**Affiliations:** Department of Internal Medicine and Endocrinology, Amphia Hospital, Breda, Netherlands

**Keywords:** clinical reasoning, endocrine disease, equation, model, morbidity

## Abstract

Most endocrine diseases are characterized by an inappropriately high or inappropriately low concentration of a certain hormone in the blood, but the relationship between the biochemical profile and morbidity of a patient with an endocrine disease cannot be reduced to a single deviation of a hormone concentration from a pre-defined reference range. Some patients hardly experience any symptoms, despite profound biochemical disturbances, whereas others seem disproportionately afflicted by measured plasma hormone concentrations which are seemingly normal. Rather than merely evaluating to what extent a plasma hormone concentration deviates from a pre-defined upper or lower limit, the dynamics and kinetics of this particular hormone should be considered with a special emphasis on its rate of change, which tends to correspond more closely to the perceived symptom severity than a single, static blood value: the “delta effect”. In this article, this phenomenon is illustrated by three relatable patient cases. A novel, clinically applicable equation is derived to describe this “delta effect” in a straightforward fashion, and to serve as a reminder that the symptoms of an endocrine disease should not be simply equated with an abnormal plasma hormone concentration, but that a kinetic (rather than static) interpretation of these blood values is necessary for adequate treatment and care. Hopefully, the presented model can help to shape clinical reasoning in both those experienced and unexperienced in the field of Endocrinology.

## 1 Introduction

Most endocrine diseases are characterized by an inappropriately high or inappropriately low concentration of a certain hormone in the blood, and many of their symptoms are the result of either over- or understimulation of this hormone’s target cells in the human body: this constitutes a dogma in Endocrinology (1–3). However, as any experienced endocrinologist knows, the relationship between the biochemical profile and morbidity of a patient with an endocrine disease is far from straightforward, and it cannot be reduced to a single deviation of a hormone concentration from a pre-defined reference range (1, 3). Some patients hardly experience any symptoms, despite profound biochemical disturbances, whereas others seem disproportionately afflicted by measured plasma hormone or metabolite concentrations which are seemingly normal (1, 3). A well-known example from diabetes care related to this last category is the “pseudohypoglycemia”, where a patient experiences hypoglycemia symptoms despite normoglycemia (4, 5). Numerous other existing clinical examples indicate that the clinical-biochemical link is not a straightforward one (1, 3). A comprehensive description of this complex relationship could help to better understand what determines the morbidity in endocrine diseases. In this respect, an intuitive, but relatively overlooked phenomenon in Endocrinology –which has not been explicitly defined, and which could be named the “delta effect”– is worth considering: rather than merely evaluating to what extent a plasma hormone concentration deviates from a pre-defined upper or lower limit, the dynamics and kinetics of this particular hormone should be considered in a broader sense, with an emphasis on its rate of change.

In the section below, three brief clinical case examples from our clinic will be discussed to highlight the pitfalls of the abovementioned traditional doctrine in Endocrinology, and a suggestion will be made for an expansion in the form of a qualitative (rather than quantitative) clinical equation. The goal of this formula is not an exact calculation, but rather a means to quickly evaluate the relevant parameters in understanding a patient’s clinical presentation. While many endocrinologists will intuitively recognize this phenomenon, a formal generalization and definition of this “clinical gut-feeling” is lacking, and seems overdue.

## 2 Patient case descriptions

Patient A, a 31-year-old female with no relevant medical history, was seen at our outpatient clinic with a recently diagnosed viral thyroiditis with a plasma free thyroxine (fT_4_) concentration of 51 pmol/L (reference: 10–22 pmol/L), and a TSH concentration of<0.01 mU/L (reference: 0.5-5.0 mU/L). Except for minor complaints of restlessness, she did not report any symptoms. After two weeks, her blood test was repeated. Her plasma fT_4_ concentration has spontaneously dropped to 15.5 pmol/L, and her TSH concentration was still<0.01 mU/L, indicating thyroiditis in resolution. During her consultation, the patient mentions feeling tired all the time, and complains of “brain fog”, weight gain, and hair loss. Her blood test results were monitored over the next few months. After approximately nine weeks, spontaneous euthyroidism was achieved, after a short period of subclinical hypothyroidism, and her symptoms had abated.

Patient B, a 38-year-old female with a medical history of primary lymphocytic hypophysitis with secondary adrenal insufficiency, for which she has been using increasing amounts of hydrocortisone over the years, visited the outpatient clinic. Because of persistent fatigue, for which no other somatic cause had been found after extensive evaluation, she had doubled the dose of her glucocorticosteroids without discussing this with her physician, and has been using twice the prescribed amount for over four months. At the moment of consultation, she shows clear signs of an exogenous Cushing’s syndrome (i.e., a “full-moon face”, and a “buffalo hump”), but still complains of fatigue and malaise shortly after taking her hydrocortisone, reminding her of hypocortisolism, and refuses to lower her dosage. A diurnal salivary cortisol curve shows strongly elevated cortisol peaks after the ingestion of hydrocortisone, with a steep decrease afterwards to normal values, indicating rapid cortisol metabolism. This patient was switched to modified release hydrocortisone and a single dose of regular hydrocortisone in the afternoon, with a significant improvement of her symptoms despite a lower cumulative dosage.

Patient C, an 18-year-old female with a mild cognitive impairment as a result of 15q13 duplication syndrome presents to our outpatient clinic with a profound primary hypothyroidism with a plasma fT_4_ concentration of 1.0 pmol/L (reference: 10–22 pmol/L), and a TSH concentration of 256 mU/L (reference: 0.5-5.0 mU/L), which was found coincidentally the week before by her pediatrician during a final blood test before her transition from pediatric to adult medical care. Her anti-TPO titer was clearly elevated, suggesting a diagnosis of Hashimoto’s thyroiditis. The patient claimed she felt fine, which was confirmed by her parents, and experienced no symptoms at all. Levothyroxine was started at an initial dose of 50 micrograms once daily, but after two weeks, the patient and her parents asked to discontinue this medication because of frequent palpitations, increased anxiety, excessive chattiness, and sleeplessness. Her new blood test shows a fT_4_ concentration of 11.8 pmol/L, and a TSH concentration of 55.4 mU/L, despite several thyrotoxic symptoms.

## 3 The “delta effect”: a mathematical model derivation

For the majority of endocrine diseases, which result from the (lack of) interaction of a certain hormone or ligand with its cellular receptor, a Weber-Fechner-like concentration-response curve can reasonably be assumed for the relationship between a patient’s biochemical profile with regard to this specific ligand (i.e., the “chemotype” C), and his or her clinical profile (i.e., “phenotype” P) (6–9) The clinical phenotype or condition can broadly be defined as the combination of observable characteristics, and physiological parameters at a single point in time: the clinical *status praesens* (10). For the sake of this model, it will be assumed that an abnormal concentration of a single hormone of interest is the main determinant of a specific endocrine disease (although this is sometimes an oversimplification of the more complex clinical reality). Mathematically, this can be described in a straightforward fashion by Equation 1, a slightly rewritten, first-order differential equation (11):

(1) $$dP=k\cdot \frac{dC}{C}$$

**Figure 1:**
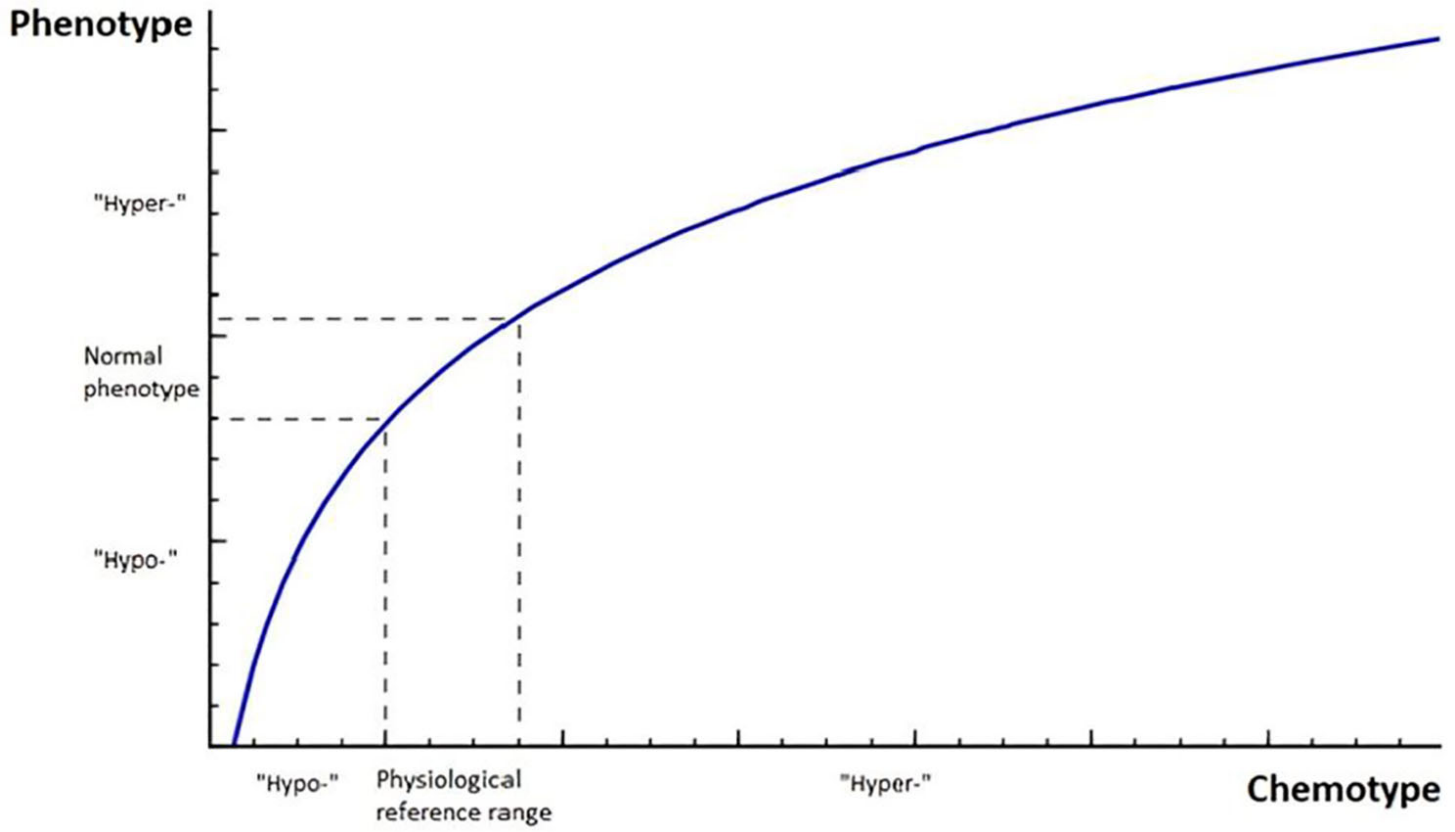
The schematic relationship between the concentration of a hormone of interest (chemotype) and the clinical phenotype, as described in the main text (Equation 2), follows a logarithmic pattern. The flattening of the curve reflects as an increased hormone receptor saturation. Note that this representation is *not* time-dependent, as opposed to its first derivative with respect to time in Equation 3 and the final Equation 4. Also note that the shoulder of the curve (with the maximum curvature) corresponds to the physiological reference range for a certain hormone concentration, and that this range marks the turning point between a “hypo-” and “hyper-” chemo- and phenotype. Note that the curve above does not intersect with the Y-axis, because the concentration of a hormone of interest cannot be 0.

Which relates the absolute change in phenotype to the relative change in chemotype, with k being the proportionality constant, which represents the individual patient’s characteristics, such as his or her receptor sensitivity, age, sex, comorbidity, and general health (1, 3). By integration, this equation produces an intuitive logarithmic result for the abovementioned concentration-response curve (**Figure 1**) (11):

(2) P=k·ln(C)+K

With K being the dimensionless constant of integration. It can easily be seen in **Figure 1** that a change in a hormone of interest’s plasma concentration produces a relatively large change in a patient’s clinical phenotype in the shoulder of the curve (reflecting this hormone’s physiological reference range at this curve’s maximum curvature), but an ever-smaller change at very high, supraphysiological concentrations due to receptor saturation (as expressed mathematically by the natural logarithm, with a limit of its curvature function for x→∞ of approximately 0) (6, 7, 11). For instance, a rise of the plasma fT_4_ concentration from 15 to 30 pmol/L (a 100% increase) will often produce tachycardia, and diaphoresis, whereas the heart rate and sweat rate will not increase comparably when this plasma thyroxin level increases from 30 to 45 pmol/L (a 50% increase), even though the absolute increment (i.e., 15 pmol/L) is the same. While it is tempting to simply state that “phenotype equals logarithm of chemotype” for an endocrine disease, it would be an oversimplification to equate a clinical phenotype with a patient’s symptoms or morbidity. As mentioned in the introduction section, not only the hormone concentration increment ( ΔC), but also the time increment ( Δt) in which this change occurs needs to be considered. If the abovementioned hyperthyroidism develops very gradually over the course of many months or even years, this patient will most likely not experience any significant symptoms (analogous to Friedrich Goltz’ questionable experiment of “the frog in boiling water” from 1869, where it was argued that a frog will not jump out of hot water as long as it is heated sufficiently slowly) (12, 13). This patient will have adapted to this gradual hormonal change: the cells will have adapted by either up- or downregulation of their hormone receptor expression, the endocrine system will have adapted by secretion of counter-regulatory hormones (e.g., glucagon negating excessive insulin release), the autonomic nervous system will have responded appropriately, and the person will have adapted psychologically (13). All of these adaptation processes to restore homeostasis take time (13). When the chemotype of a patient changes so rapidly that this adaptation cannot keep up with the change, clinical symptoms seem to occur or increase (even when the biochemical profile of a patient is within its physiological reference range, such as with patients A-C). This is certainly not to say that the magnitude of change in a hormone’s plasma concentration is irrelevant, but the rate of relative change seems to be a more accurate and a more intuitive determinant of the symptom severity than the change itself. Therefore, Equation 2 needs to be rewritten in a time-dependent fashion to express a patient’s symptom severity ( S):

(3) $$S\left(t\right)=\frac{dP\left(t\right)}{dt}=k\cdot \frac{d\ln\left(C\left(t\right)\right)}{dt}$$

If a situation is considered where a hormone of interest’s plasma concentration is changed from $C_{1}$ to $C_{2}$ over a period of time of $\Delta t=t_{2}-t_{1}$, then the average symptom burden ( $\bar{S}\left(t\right)$) during this interval, or morbidity ( M), can be expressed as follows by integration and the mean value theorem for integrals:

(4) $$M=\bar{S}\left(t\right)=k\cdot \frac{\Delta \ln\left(C\right)}{\Delta t}$$

This result could be considered a straightforward, but intuitive representation of the previously coined “delta effect”. The governing equation above incorporates the three principal determinants of morbidity in a patient with a typical endocrine disease, namely: the patient and hormone receptor characteristics (as expressed by a combined parameter k), the relative change of a hormone of interest’s plasma concentration (as expressed by Δln(C)), and the time in which this change occurs (as expressed by Δt).The natural logarithm in the numerator mathematically reflects the physiological flattening the concentration-response curve due to the receptor saturation with its ligand (6, 7). Any difference in experienced symptoms between two arbitrary patients resulting from an identical change in a plasma hormone concentration should be explained by a difference in the rate of relative change and/or by individual patient characteristics, as reflected by k. This is also mathematically evident: assuming first-order kinetics, which is true for the vast majority of hormones, integrating over the interval in which this change occurs $\Delta t=t_{2}-t_{1}$ shows that the average plasma hormone concentration $\bar{C}$ between two concentrations $C_{1}$ and $C_{2}$ is independent of this interval Δt (i.e., these two arbitrary patients are not only exposed to the same $\Delta C=C_{2}-C_{1}$, but also to the same $\bar{C}$, regardless of the interval duration), as described by Equation 5:

(5) $$\bar{C}=\frac{C_{2}-C_{1}}{\ln\left(C_{2}/C_{1}\right)}=\frac{\Delta C}{\Delta \ln\left(C\right)}$$

## 4 Discussion

Every endocrinologist recognizes patients with symptoms or complaints that simply do not correspond to the obtained blood test results; hormone levels may look better than before, whereas a patient may feel significantly worse. This is a vexing clinical problem, which can –at least partly– be solved by appreciating the static nature of a single blood test, as opposed to the dynamic nature of an endocrine disease, and the human body as a whole. In the previous section, a straightforward, clinically applicable model has been presented to describe the key elements of morbidity in a “typical” endocrine disease. Its aim is not to perform exact calculations, but rather to get a qualitative understanding of how several relevant factors contribute to the overall symptom burden of an endocrine disease. At the very least, the proposed equation is a gentle reminder that the symptoms of an endocrine disease should not be simply equated with an abnormal plasma hormone concentration, and that a kinetic (rather than static) interpretation of these blood values is necessary for adequate treatment and care: the delta effect. This becomes even more intuitive when realizing that the plasma concentration of a certain hormone of interest –while still the backbone of diagnostics in Endocrinology– is an imperfect, crude measure of the actual effective hormone concentration on a cellular level (and thus the biological effect), as postulated by the “free hormone hypothesis” (3, 14). On the other hand, the *trend* (i.e., the increase or decrease) of a certain hormone in the blood is very likely mirrored on a cellular level due to the body’s tendency to maintain hormonal association-dissociation equilibria according to Le Châtelier’s principle (6, 7). The physiological reference range for the concentration of a hormone of interest, as reported by the clinical laboratory, intuitively corresponds to the shoulder of the curve of Equation 2 (**Figure 1**), which explains why the presented model is also well-suited to describe endocrine morbidity in the traditional sense: a rapid increase (or decrease) of the concentration of a hormone of interest above (or below) the reference range produces characteristic symptoms, as every endocrinologist recognizes. The same principles apply here. The derivation of this equation is based on two main pillars, namely: the Weber-Fechner-like relationship between the “chemotype” and the “phenotype”, and the incorporation of time-dependency (11). While the abovementioned equation presupposes the interaction of a certain hormone of interest with its receptor, the same line of thinking could be extended to other closely related biochemical disturbances in the human body, where there may not be a clear ligand-receptor interaction, but where the same chemotype-phenotype relationship applies. Examples are rapidly fluctuating plasma glucose levels (e.g., “pseudohypoglycemia”), and many electrolyte disturbances as a result of an endocrine disease (4, 5). Examples of the latter are patients who have undergone a parathyroidectomy as a treatment for primary hyperparathyroidism, who complain of paresthesia, and muscle cramps post-operatively, while their plasma calcium concentrations are still well above the upper limit of normal, and the (often neurological) *sequalae* of an overly rapid increase or decrease of the plasma sodium concentration in a chronic dysnatremia (15).

Patients A-C all experience considerable symptoms, despite plasma concentrations of their hormones of interest being perfectly within the reported reference range. In some cases, there even is a paradoxical discrepancy between the patient’s reported symptoms, and his or her biochemical profile (e.g., patient C, who experiences thyrotoxic symptoms despite a normal fT_4_ and elevated TSH). This inconvenient paradox could tempt a physician to dismiss these symptoms. However, if Equation 4 is applied to the patients A-C, it can be seen that the large value for Δln(C) and the small value for Δt lead to significant morbidity M in all three patients, despite “normal” blood test values, according to Equation 6:

(6) $$M↑↑=k\cdot \frac{\Delta \ln\left(C\right)↑}{\Delta t↓}$$

The extent to which adaptation to an endocrine disturbance has already taken place seems to be an important determinant of the symptom severity. As a general rule, the longer such a disturbance has existed, the more likely that the body has adapted to it on a cellular, systemic, and psychological level, and the less likely that this disturbance produces any significant symptoms (12). Whenever this chronically adapted system is subjected to a relatively large and relatively rapid change, prohibiting sufficient adaptation (which takes time), the patient experiences symptoms, regardless of the numerical blood test values. As seen before, these values can be perfectly within range at a single point in time, whereas the endocrine system is in severe disequilibrium.

The constant k deserves some special consideration. It is determined by –among many other factors, such as general health– individual hormone receptor expression and sensitivity, which exhibits a strong interindividual variability (e.g., patient B already shows clear Cushingoid stigmata at a hydrocortisone dosage which would not normally produce such a phenotype) (16, 17). Individual receptor expression and sensitivity is also a major, but often neglected, determinant of a patient’s clinical phenotype as a result of a certain biochemical profile, and mathematically links a measured rate of biochemical change to the experienced symptom severity, as expressed by the presented equation. While the expression of a certain hormone receptor is largely regulated by acquired factors, its sensitivity is determined genetically (more extreme cases of hormone receptor dysfunction can even be classified as a separate disorder, such as the thyroid hormone resistance syndrome due to a “loss-of-function” mutation in the *THRB*-gene) (6, 7, 18). Unfortunately, there are currently no reliable, clinically available techniques to measure or quantify hormone receptor sensitivity, but this important factor should nevertheless be taken into consideration while evaluating a patient’s symptoms.

In conclusion, an attempt has been made to mathematically formalize the endocrinologist’s clinical intuition in this article. A clinically applicable model is derived on theoretical grounds, which aims to aid in understanding and qualitatively analyzing morbidity in a patient with a “typical” endocrine disease. While its biochemical nature makes Endocrinology the ideal example for the application of the presented equation, its application could theoretically be extended to other medical fields. It goes without saying that the human body can never be reduced to a set of equations, but hopefully this model serves as reminder for clinical endocrinologists that there is always more truth to a patient’s symptom experience than to a numerical result of a blood test. Sometimes a disease hides in plain sight. As the renowned internist William Osler once said: “listen to your patient; he is telling you the diagnosis.”

## Data Availability

The original contributions presented in the study are included in the article/supplementary material. Further inquiries can be directed to the corresponding author.
